# Influenza A Virus–Host Protein Interactions Control Viral Pathogenesis

**DOI:** 10.3390/ijms18081673

**Published:** 2017-08-01

**Authors:** Mengmeng Zhao, Lingyan Wang, Shitao Li

**Affiliations:** 156 McElroy Hall, Department of Physiological Sciences, Oklahoma State University, Stillwater, OK 74078, USA; mengmeng.zhao@okstate.edu (M.Z.); lingyan.wang@okstate.edu (L.W.)

**Keywords:** flu, protein interactome, virus–host interactions, host defense, virulence

## Abstract

The influenza A virus (IAV), a member of the Orthomyxoviridae family, is a highly transmissible respiratory pathogen and represents a continued threat to global health with considerable economic and social impact. IAV is a zoonotic virus that comprises a plethora of strains with different pathogenic profiles. The different outcomes of viral pathogenesis are dependent on the engagement between the virus and the host cellular protein interaction network. The interactions may facilitate virus hijacking of host molecular machinery to fulfill the viral life cycle or trigger host immune defense to eliminate the virus. In recent years, much effort has been made to discover the virus–host protein interactions and understand the underlying mechanisms. In this paper, we review the recent advances in our understanding of IAV–host interactions and how these interactions contribute to host defense and viral pathogenesis.

## 1. Introduction

Influenza A virus (IAV) is a human respiratory pathogen that causes seasonal epidemics and occasional global pandemics with devastating levels of morbidity and mortality [1,2,3,4]. IAV is an enveloped virus that belongs to the Orthomyxoviridae family [5]. On the lipid bilayer of virus particle, there are two abundant membrane proteins, hemagglutinin (HA) and neuraminidase (NA), and a few copies of matrix protein 2 (M2). Beneath the lipid membrane is matrix protein 1 (M1), which forms a shell and gives strength and rigidity to the lipid envelope. Within the core of the virion are eight segments of negative-sense single-stranded RNA genome encoding at least 11 proteins. Each RNA segment is associated with multiple copies of nucleoproteins (NP) and a small number of heterotrimeric RNA-dependent RNA polymerase complexes that comprise polymerase acidic (PA), polymerase basic 1 (PB1) and polymerase basic 2 (PB2), which together form the ribonucleoprotein complex (RNP).

To initiate infection, HA binds to sialic acid-linked glycolipids or glycoproteins on host cell surface. Subsequently, the virus ferries into cytoplasm through the endocytic pathway. In the late endosome, low pH conditions trigger conformational changes of HA and the fuse between viral and endosomal membranes, thereby forming a fusion pore ready for the release of viral RNP into cytosol. At the same time, M2 functions as a viral ion channel protein to assist RNP dissociation from M1 proteins. Once RNP is exposed in the cytoplasm, it migrates to the nucleus, where the viral genome serves as a template for synthesis of mRNA and complementary RNA; the latter functions as a template for genomic RNA replication. The newly synthesized viral RNPs (vRNPs) are exported to the cytoplasm with the assistance of M1 and non-structural protein 2 (NS2). The vRNPs are assembled with HA, NA, M1, and M2 under the cell membrane. Finally, the budding viral progenies are freed from the infected host cell by the cleavage of NA on the sialic acid.

IAV is a zoonotic virus that also infects many other species, such as wild waterfowl, poultry, and pigs. IAV is subtyped based on genetic and antigenic differences in the HA and NA glycoproteins. Currently, there are 18 HA subtypes and 11 NA subtypes have been detected in the wild [6]. Although HA and NA are critical for viral entry and egress, other viral proteins express in host cells and encounter the cellular protein network, which also determines the virus fate in host and contributes to viral pathogenesis. This review focuses on recent advances of the interactions between IAV proteins and host cellular factors.

## 2. Non-Structural Protein 1 (NS1)

NS1 is a late expressing viral protein and not packed in the IAV virion. NS1 has been extensively studied in recent years due to its multifunctional trait [1,2,3,4,7], which involves host innate immune defense, host and viral mRNA expression, apoptosis, viral RNA splicing and morphogenesis. Most of these NS1 functions are dependent on the protein interaction with host factors. For example, NS1 binds to the cellular double-stranded RNA sensors such as protein kinase R (PKR) [8,9,10], retinoic-acid inducible gene I (RIG-I) [11] and 2′–5′ oligoadenylate synthetase [12] directly or indirectly through viral RNA, thereby preventing these sensors from activating innate immune responses including type I interferon expression. NS1 also interacts with the regulators of these sensors, such as the protein activator EIF2AK2 ((PRKRA, also known as PACT) [13], tripartite motif containing 25 (TRIM25) [14], and NF90 [15]. TRIM25 is an E3 ligase that ubiquitinates RIG-I, which is critical for RIG-I activation [16]. NS1 interacts with the coiled-coil domain of TRIM25, thus blocking TRIM25-mediated RIG-I activation [14]. Additionally, NS1 interacts with IKKa/b to impair the nuclear Factor kappa-light-chain-enhancer of activated B cells (NF-κB) signaling pathway [17], whereas the interaction with RNA-associated protein 55 (RAP55) counteracts the stress granule-mediated antiviral activity [18].

In addition to subversion of the innate immune signaling pathway, NS1 blocks host defense by suppression of cellular mRNA nuclear export and expression, such as the expression of type I interferon genes. NS1 interacts with poly(A)-binding protein II (PABPII) [19] and host mRNA export machinery consisting of nuclear rna export factor 1 (NXF1), ribonucleic acid export 1 (RAE1) and E1B-55K-associated protein 5 (E1B-AP5) [20]. These interactions block the nuclear export of fully processed host mRNAs. NS1 also interacts with the 30 kDa subunit of cleavage and polyadenylation specificity factor (CPSF30), which prevents CPSF30 from binding cellular pre-mRNAs, thereby inhibiting the cleavage and polyadenylation of host mRNAs [21,22,23]. Several residues of NS1 are required for CPSF30 interaction, including F103 and M106 [24]. F103 and M106 are conserved among most human IAVs, but are mutated in some strains, such as PR8 [24]. Consistently, the NS1 of PR8 IAV fails to interact with CPSF30 [25,26]. More importantly, L103F and I106M mutations in the NS1 of H5N1 viruses increase NS1 interaction with CPSF30, thereby suppressing type I interferon expression and facilitating viral replication [25]. These studies set excellent examples of how the influenza–host protein interaction controls and host defense and virulence.

It is also well established that NS1 activates the phosphoinositide 3-kinase (PI3K)/AKT serine/threonine kinase 1 (AKT) pathway, which delays virus-induced apoptosis and provides sufficient time for virus replication [27,28,29,30]. NS1 binds directly to the p85 beta regulatory isoform of PI3K [28,30,31,32,33,34]. Recent studies also found that the NS1 activate PI3K by binding the N-terminal SH3 domains of Crk and/or CrkL [29,35,36]. Additionally, NS1 has been found to interact with AKT [37] and p53 [38] to promote cell survival. On the contrary, NS1 interacts with heat hock protein 90 [39] and alpha tubulin [40] to facilitate apoptosis.

Furthermore, NS1 interacts with host proteins containing a PSD-95/Discs-large/ZO-1 (PDZ) domain through the C-terminal motif consisting of “ESEV” in avian viral isolates or “RSEV” in human viral isolates [41,42,43,44]. The PDZ binding motif of NS1 functions as a virulence determinant, which is cell type and species-dependent [41,42,44]. When the PDZ ligand motif of pandemic 1918 and H5N1 highly pathogenic avian influenza viruses was added to the NS1, the recombinant WSN influenza became more pathogenic [41]. Several PDZ domain containing proteins have been found to interact with NS1, including the PDZ and LIM domain 2 (PDlim2) [45], postsynaptic density protein 95 (PSD-95) [46], Scribble [47,48], discs large homolog 1 (Dlg1) [47,48], membrane associated guanylate kinase, ww and pdz domain containing 1 (MAGI-1) [47,48], MAGI-2 [47,48], and MAGI-3 [47,48]. The interactions with these PDZ proteins contribute to diverse biological function, such as interferon expression [49], cellular tight junction [50] and nitric oxide production [46].

Lastly, NS1 protein utilizes host factors to facilitate viral replication, RNA splicing and viral morphogenesis. For example, NS1 enhances the translation of viral mRNA through interaction with eIF4GI and PABPI [51]. NS1 also interacts with RNA helicase A [52] and DEAD-box RNA helicase 21 (DDX21) [53] to regulate viral replication and gene expression. NS1-binding protein (NS1-BP) forms a complex with heterogeneous nuclear ribonucleoproteins and regulates viral M1 RNA splicing [54]. In the late stages of the viral replication cycle, the NS1 protein may be involved in morphogenesis of the virus particles through binding with the Staufen protein [55,56].

## 3. Non-Structural Protein 2 (NS2)

The non-structural protein NS2 (also known as nuclear export protein [57]) regulates viral genome transcription/replication [58,59], nuclear export of vRNP complexes [60,61,62,63,64], and virus budding [59,65]. Several studies have shown that NS2 acts as an adaptor to mediate the progeny vRNP nuclear export by forming protein complex with chromosome region maintenance 1 (CRM1) and M1 [60,61,62,63,64]. The N terminal nuclear export signal (NES) of NS2 interacts with the CRM1 while the C terminus binds the M1 protein, thereby forming the CRM1–NS2–M1–vRNP nuclear export complex [57,60,61,66,67]. Recently, the chromodomain helicase DNA binding protein 3 (CHD3) was found to interact with the NES of NS2 and facilitate vRNP export [68]. vRNP is exported through the nuclear pore complex (NPC). The nucleoporin 98 (Nup98), a NPC component, was identified as a NS2 interacting protein; however, the consequence of the interaction is not well elucidated [69].

In addition, it has been reported that NS2 interacts with the plasma membrane-associated F1Fo ATPase, which is critical for influenza virion formation and budding [65]. Recently, NS2 has been found to interact with aminoacyl-tRNA synthetase (AIMP2) [70]. The interaction of NS2 with AIMP2 facilitates the switch of the M1 modification from ubiquitination to SUMOylation, thus increasing viral replication [70].

## 4. M1

The M1 protein of IAV mediates nuclear export of viral ribonucleoproteins [71], inhibits viral transcription [72,73], participates virus assembly and budding [74], and modulates host defense [75]. Several host factors have been found to involve some of these processes by interacting with M1. For example, the cellular receptor of activated C kinase 1 (RBCK1) interacts with M1 and might bridge protein kinase C (PKC) to the proximity, which subsequently phosphorylates the M1 [76]. Extracellular signal-regulated kinase (ERK) has also been shown to phosphorylate M1 in vitro [77]. Cyclophilin A interacts with M1 and impairs early virus replication [78]. M1 also interacts with complement C1qA, which blocks the classical complement pathway by disruption of the interaction between C1qA and heat-aggregated IgG [75]. In addition, the globular domain of the histone octamer [79], heat-shock protein 70 [80], cytoskeletal elements [81], and caspases [82] have been shown to associate with M1, but the underlying mechanisms are not clear.

## 5. M2

M2 functions as a proton-selective ion channel that conducts protons from the acidified endosomes into the interior of the virus particle to allow the release of the vRNPs into the cytoplasm [83,84]. It is also known that M2 regulates IAV assembly and budding [85,86,87,88,89]. Recently, several host proteins have been found to associate with M2 and regulates viral protein transport. The Golgi-specific brefeldin A resistant guanine nucleotide exchange factor 1(GBF1) associates with M2, which is crucial for viral protein transport to the plasma membrane [90]. The putative ubiquitin ligase UBR4 also associates with the viral M2 protein and promotes apical transport of viral proteins [91]. The transport protein particle complex 6A delta (TRAPPC6AΔ) was reported to interact with M2 [92]. The interaction positively regulates viral replication in vitro by modulating M2 trafficking to the plasma membrane [92]. Additionally, the cytoplasmic domain of influenza M2 protein interacts with caveolin-1 [93], but whether this interaction affects viral trafficking is not known.

## 6. Nucleoprotein (NP)

NP is the most abundantly expressed viral protein during the course of infection and plays multiple roles in viral life cycle, including nuclear transport of vRNP, and transcription and replication of the viral genome. Much effort has been made to elucidate how host factors assist NP nuclear transport. For example, karyopherin subunit α 1 (KPNA1, also known as NPI-1 and SRP-1) and α 2 (KPNA2, also known as NPI-2 and Rch-1) are the first identified NP-interacting proteins that facilitate NP nuclear import [94,95,96]. Recently, the nuclear localization signal of NP has been show to interact with importin 5 (IPO5, also known as KPNB3) for nuclear import [97]. Similarly, NP also binds CRM1 and nuclear transport factor 2 (NTF2)-like export protein 1 (NXT1) for nuclear export [98,99].

In addition, NP exploits cellular factors through protein interaction to promote viral replication and suppress host innate immune response. The DExD-box helicase 39B (DDX39B, also known as BAT1, NPI-5, and UAP56) and high mobility group box 1 (HMGB1) have been reported to interact with NP, which promotes viral RNA synthesis and viral replication [100,101]. NP also exploits heat shock protein 40 (Hsp40) to inhibit PKR activation [102]. Additionally, the Ccr4-Not transcription complex subunit 4 (CNOT4) binds and ubiquitinates NP to promote viral RNA replication [103].

On the contrary, host elicits intrinsic and innate immune responses by direct interaction with the viral NP protein. For example, the nuclear MxA suppresses the influenza virus transcription by interacting with NP [104]. RuvB-like protein 2 interferes with NP oligomerization, thereby disrupting vRNP assembly [105]. DEAD-box RNA helicase 3 (DDX3) interacts with NP and exerts antiviral function through regulation of stress granule formation [106]. Interferon-stimulated exonuclease gene 20 interacts with NP and inhibits viral protein expression [107]. The E3 ligase TRIM22 was reported to ubiquitinate NP and target it for protein degradation, thereby inhibiting viral replication [108].

## 7. PB1 Frame 2 (PB1-F2)

PB1-F2 is a small non-structural viral protein, encoded by an alternate (+1) reading frame within the PB1 gene [109,110,111]. PB1-F2 localizes to mitochondrial membranes and induces cell death [109]. PB1-F2 interacts with the mitochondrial proteins adenine nucleotide translocator 3 (ANT3) and voltage-dependent anion channel 1 (VDAC-1) [110], which destabilizes mitochondrial membrane and subsequently promotes apoptosis [110]. Conversely, the nucleotide-binding oligomerization domain-like receptor (NLRX1) binds to PB1-F2 and prevents mitochondrial apoptosis [112].

Although the NS1 is thought of as the key viral protein that inhibits host innate immunity, recent studies revealed that PB1-F2 also blocks antiviral innate responses [113,114]. However, PB1-F2 counteracts host defense in a different way from NS1. NS1 exerts a global inhibitory effect on host gene expression including interferon expression whereas PB1-F2 specifically subverts the innate immune signaling pathway. PB1-F2 interacts with IKKb and inhibits NF-κB signaling [115]. PB1-F2 also binds to the mitochondrial antiviral signaling protein (MAVS), the key molecule on mitochondria of RIG-I signaling pathway and reduces mitochondrial membrane potential [113,114], thus indirectly inhibiting viral RNA-activated RIG-I signaling pathway.

## 8. Polymerase Complex

Replication and transcription of these IAV segments are catalyzed by a heterotrimeric RNA-dependent RNA polymerase complex, which consists of an acidic subunit (PA) and two basic subunits (PB1 and PB2) [116,117]. Replication and transcription of the IAV genome are not sufficient when only viral components are present, suggesting cellular factor(s) is exploited to fulfill the process [118]. Using an in vitro RNA synthesis assay in combined with fractionation of cell nuclear extracts, Nagata’s group identified BAT1 and heat shock protein 90 (Hsp90) as the cellular factors required for viral replication [100,119]. BAT1 interacts with NP while Hsp90 associates with PB2 monomers or PB2/PB1 heterodimers [119,120]. Hsp90 is able to stimulate viral RNA polymerase activity and is involved in the trimeric polymerase complex assembly and nuclear import of the virus polymerase subunits [119,120]. Recently, acidic nuclear phosphoprotein 32 family member A (ANP32A, also known as pp32) and B (ANP32B, also known as APRIL) were identified as viral polymerase interacting proteins that regulates influenza virus RNA synthesis from complementary RNA (cRNA) [121]. ANP32A has also been identified as a key cellular co-factor that dictates host specificity. The chicken ANP32A is required for the sufficient replication of avian flu in human cells [122]. Viruses encoding an avian-origin polymerase replicate poorly in mammalian cells and are attenuated in mammalian hosts [123], suggesting the existing of unknown host determinants. ANP32A is one of such candidates because the 33-amino acid insert of chicken ANP32A is missing in mammalian counterparts and required for avian influenza polymerase activity [122]. The exciting finding also raises new questions that remain to be answered—for example, how the 33-amino acid insert of ANP32A discriminates the viral polymerases of avian and mammalian influenza viruses, whether it increases the binding affinity with avian influenza polymerase and how it regulates polymerase activity.

Several other host cellular proteins were also found to interact with influenza virus polymerase and promote viral replication, including cyclin-dependent kinase 9 [124], protein phosphatase 6 [125], C14orf166 (also known as CLE) [126,127], heat shock protein 40 [128], heat shock protein 70 [129], and acetyl-CoA [130]. In addition, the DNA replication regulator and spliceosomal factor SMU-1 is recruited by IAV polymerase to control viral mRNA splicing [131]. Recently, influenza viral polymerase complex was found to recruit the nuclear RNA exosome complex for cap-snatching [132]. The nuclear RNA exosome coordinates the initial steps of viral transcription with RNAPII at host promoters. Influenza viral polymerase complex co-opts the nuclear RNA exosome complex and cellular RNAs en route to 3′ end degradation, thus snatching host 5’ cap as a primer for the initiation of viral mRNA synthesis [132]. RNA exosome and its cofactors are known to recognize and degrade different viral RNAs [133], but how influenza protects its RNA from RNA exosome needs further investigation.

It has been reported that PB2 enters the nucleus by interacting with importin isoforms [95,132,134], and the interaction between PB2 and different importin isoforms governs the cell tropism of IAV [95,132,134,135,136]. Recently, one study reported that cellular protein HAX1 interacts with the influenza virus PA polymerase subunit and impedes its nuclear translocation [137].

Since the activity of RNA-dependent polymerases is distinct from enzymes found in host cells, these viral proteins are potential targets of host immune defense. Recently, our lab found that two host factors, tripartite motif protein 32 (TRIM32) and plakophilin 2 (PKP2), interact with PB1 and impede viral polymerase activity by two distinct mechanisms [26,138]. TRIM32 is an E3 ubiquitin ligase and targets viral PB1 protein for ubiquitination and subsequent protein degradation [138]. PKP2 competes with PB2 for PB1 binding and impairs the integrity of viral polymerase complexes, thereby limiting viral polymerase activity [26]. DDX21 RNA helicase also restricts influenza A virus by binding PB1 and inhibiting polymerase assembly [53]. Conversely, viral polymerase PB2 has been found to subvert host innate immunity. A small portion of PB2 expresses in mitochondria and inhibits host interferon production by interacting with MAVS, a key signaling molecule of RIG-I-mediated innate immunity pathway [139,140].

## 9. Systematic Map of IAV–Host Protein Interactome

**Figure 1:**
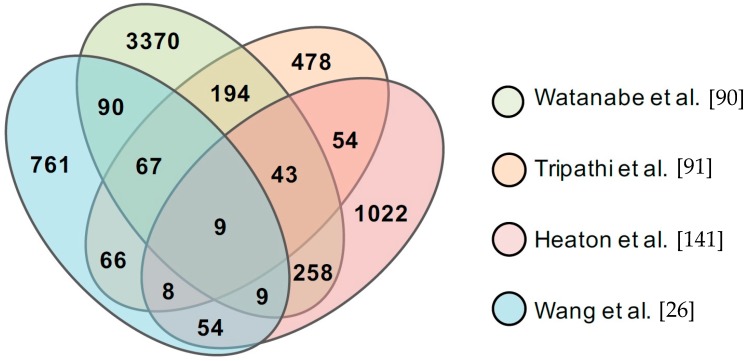
Venn diagrams illustrating the number of common host factors identified in different studies.

The identification of interactions between viral and host cellular proteins has provided major insights into influenza research, and recent advances in technology now allow for systematically mapping influenza–host protein interaction network. Several publications have reported a cohort of cellular factors that interact with IAV proteins by large-scale proteomics or yeast two-hybrid [26,90,91,141,142,143,144]. We will compare three recent comprehensive studies using a single influenza strain [90,91,141] and our own study using multiple strains [26]. All four studies adopted affinity purification coupled with mass spectrometry with different features (Table 1). Surprisingly, the overlap of specific protein interactions identified by these studies is relatively low (Figure 1). Reasons for the discrepancy could be the false positives and false negatives endemic to the systematic approach, or other conditions such as virus strain, cell type, and statistical algorithm (Table 1). Nonetheless, a total of 136 common protein interactions have been found in at least three studies (Figure 1 and Supplementary Table S1), which are worthy of further investigation. For example, all four studies found that M2 interacts with two subunits of the transport protein particle complex (TRAPPC), TRAPPC5 and TRAPPC9 (Supplementary Table S1), suggesting a role of TRAPPC in the regulation of influenza M2. Indeed, a recent study found that TRAPPC is required for M2 trafficking to the apical plasma membrane and promotes viral replication [92], which is an excellent example of how we gain insight from systematic protein interaction data.

Although these studies provide unprecedented molecular insights into host–pathogen interactions, most of the interactions have not been validated using small-scale and more directed experiments. Furthermore, as with the nature of systematic study, these databases may not represent a complete interaction network; in fact, a few well-established interactions are missing in some of these databases.

## 10. Perspectives

Influenza control strategies rely on annual immunizations and require frequent updates of the vaccine, an expensive, cumbersome, and not always foolproof process [148,149]. Furthermore, the currently antivirals are also losing effectiveness as new viral strains are often refractory to conventional treatments. Thus, there is an urgent need to find new antiviral mechanisms and develop therapeutic drugs based on these mechanisms. As reviewed in this paper, virus exploits cellular machinery by protein interactions and on the contrary, the engagement also elicits host immune defense to limit viral infection. Therefore, the potential therapeutic targets at the influenza–host interface are promising.

Most virus–host protein interactions have only been studied in a few strains that are widely used in the laboratory; however, IAV comprises a plethora of strains with different pathogenicity, transmissibility, and pandemic potential. For example, the NS1 of 2009 pandemic flu lacks all previously identified virulence markers [1,150] and little is known about the underlying mechanisms by which the NS1 of 2009 pandemic flu contributes to the outcomes of pathogenicity and pandemics. Comparative analysis of NS1 protein interactomes of different influenza strains will provide insights and clues to the answer. Furthermore, most protein interactions are identified under static conditions. Dynamic interactions under infection and pathogenic conditions are more attractive because they reflect the intrinsic signaling cascades. For example, hypoxia affects influenza virus proliferation [151,152], but how hypoxia alters influenza–host protein interactions arise not known. Thus, future research should improve our understanding of the different pathologies associated with various IAV strains, and the diverse nature of these viruses is reflected in the spectrum of interactions with host proteins. Ultimately, comprehensive knowledge of IAV–host interactions may help us decipher the therapeutic targets and the molecular mechanisms of viral pathogenesis.

## Figures and Tables

**Table 1:** Recent IAV–host protein interactomes discussed in the review.

References	Strain	Epitope	Cell Line	Viral Infection	Statistical Algorithm
Watanabe et al. [90]	WSN	FLAG at N or C terminal	HEK293	No	N/A
Tripathi et al. [91]	WSN	FLAG	HEK293	No	CompPASS [145] and MiST [146]
Heaton et al. [141]	PR8	FLAG	A549	Yes	MiST [146]
Wang et al. [26]	PR8, WSN, Aichi, NY/2009, VN/2004	FLAG at N or C terminal	HEK293	Yes	SAINT [147]

## Supplementary Materials

Supplementary materials can be found at www.mdpi.com/1422-0067/18/8/1673/s1.
